# Supplementary material for: Content-rich biological network constructed by mining PubMed abstracts
Source: BMC Bioinformatics. 2004 Oct 8;5:147. doi: 10.1186/1471-2105-5-147 (PMC528731; doi:10.1186/1471-2105-5-147)
Supplement: Additional File 2 — The original results of the above study (non-essential files are deleted to keep the file size under the limit set by BMC bioinformatics). [file 1471-2105-5-147-S2.bz2 › chilibotAdditionalFile2/dip50/80ID9033409E292/html/PAK3_RAC1.html]

 


 **PAK3** and **RAC1** 
  
Found 258 abstracts in PubMed, retrieved 50.  
 

 What does Google say? 
 PDF only 
| .edu only 

---

**Interactive relationship** (e.g. stimulation, inhibition, etc)

**Neutral relationship**- Interaction between p21  [ **PAK3** ]  activated protein kinase and Rac  [ **RAC1** ]  during differentiation of HL 60 human promyelocytic leukemia cell induced by all trans retinoic acid.  Ref: 12027902 Eur J Biochem, 2002
- p21  [ **PAK3** ]  Activated kinases are regulated by the GTPases Rac  [ **RAC1** ]  and Cdc42 and control actin dynamics and phosphorylation of the oxidase component p47.  Ref: 12189148 J Biol Chem, 2002
- Concomitant with our finding that the small GTPases Cdc42 and  **Rac1**  were activated by attachment to FN, constitutively active ca Cdc42 and ca  **Rac1**  promoted down regulation of p21  [ **PAK3** ]  CIP1.  Ref: 12052868 Mol Cell Biol, 2002
- The p21  [ **PAK3** ]  activated protein kinases are regulated by the Rho family GTPases Rac  [ **RAC1** ]  and Cdc42.  Ref: 12529371 J Biol Chem< MedlineTA>J Biol Chem, 2002
- p21  [ **PAK3** ]  activated kinase 1 PAK1 is an effector of Rac  [ **RAC1** ]  Cdc42 GTPases that has been implicated in the regulation of cytoskeletal dynamics, proliferation, and cell survival signaling.  Ref: 12522133 J Biol Chem, 2003
- Mammalian p21  [ **PAK3** ]  activated kinase 1 Pak1 is a highly conserved effector for the small GTPases Cdc42 and  **Rac1** .  Ref: 12176333 Curr Biol, 2002
- We fractionated endogenous  **Rac1**  containing protein complexes from membranes of Madin Darby canine kidney cells and identified three major complexes comprising a  **Rac1** .PAK p21  [ **PAK3** ]  activated kinase complex, and 11 S and 16 S  **Rac1**  complexes.  Ref: 12244058 J Biol Chem, 2002
- The family of p21  [ **PAK3** ]  activated kinases PAKs have been implicated in the rearrangement of actin cytoskeleton by acting downstream of the small GTPases Rac  [ **RAC1** ]  and Cdc42.  Ref: 12181358 Mol Biol Cell, 2002
- p21  [ **PAK3** ]  activated protein kinases PAK are a family of serine threonine protein kinases which are activated by binding of the p21  [ **PAK3** ]  G proteins Cdc42 or Rac  [ **RAC1** ] .  Ref: 12853446 J Biol Chem< MedlineTA>J Biol Chem, 2002
- The p21  [ **PAK3** ]  activated protein kinases Paks are serine threonine protein kinases activated by binding to Rho family small GTPases, Rac  [ **RAC1** ]  and Cdc42.  Ref: 12860998 J Biol ChemJ Biol Chem, 2002
- However, dominant negative dn Cdc42 and dn  **Rac1**  mutants blocked the anchorage induced degradation of p21  [ **PAK3** ]  CIP1, suggesting that an integrin induced Cdc42  **Rac1**  signaling pathway activates proteasomal degradation of p21  [ **PAK3** ]  CIP1.  Ref: 12052868 Mol Cell Biol, 2002
- Expression of RhoGDI, a negative regulator of  **Rac1** , inhibited secretory responses while overexpression of effectors of  **Rac1** , notably, p21  [ **PAK3** ]  activated kinase Pak1 and actin depolymerization factor ADF promoted evoked secretion.  Ref: 12754309 J Physiol< MedlineTA>J Physiol, 2002
- The ephrinA1 mediated morphological alterations correlated with inhibition of  **Rac1**  and p21  [ **PAK3** ]  activated kinase 1 PAK1 activity, and were antagonized by the expression of a constitutively active Rac  [ **RAC1** ]  mutant.  Ref: 12615978 J Cell Sci, 2003
- Surprisingly CRP did not activate the low molecular weight G protein Rac  [ **RAC1** ]  and stimulated only a small increase in activity of p21  [ **PAK3** ]  associated kinase 2, despite the fact that both proteins are regulated downstream of Vav1 in other cells.  Ref: 12411320 Blood, 2002
- The p21  [ **PAK3** ]  activated kinases are serine threonine protein kinases that bind to and, in some cases, are stimulated by activated forms of the small GTPases, Cdc42 and Rac  [ **RAC1** ] .  Ref: 11950587 Int J Biochem Cell Biol, 2002

**Non-interactive relationship** (e.g. studied together, co-existance, homology, etc.)

- 3 Soluble regulatory proteins GTPase activating protein, guanine nucleotide dissociation inhibitor, and the Rac  [ **RAC1** ]  binding domain of the target protein p21  [ **PAK3** ]  activated kinase inhibit activation of the NADPH oxidase but have no effect on electron transfer via the assembled enzyme complex.  Ref: 12186557 Biochemistry, 2002
- PAK1 p21  [ **PAK3** ]  activated kinase 1 activation is induced by C albicans, suggesting that PAK1 may also be involved in the  **Rac1**  activation of MAPK ERK.  Ref: 12511425 Blood, 2003
- Addition of PAR1 AP inhibited basal Rac  [ **RAC1** ]  activity as well as the phosphorylation of the Rac  [ **RAC1** ]  effector, p21  [ **PAK3** ]  activated kinase PAK.  Ref: 12871383 J Thromb Haemost, 2003
- 3 The Rac  [ **RAC1** ]  binding domain of p21  [ **PAK3** ]  activated kinase is a potent inhibitor of activation by the p47phox and amphiphile independent pathway while exerting a milder inhibitory effect on the canonical pathway.  Ref: 12875368 Inflammation, 2003
- Nef, a multifunctional HIV protein, activates the Vav Rac  [ **RAC1** ]  p21  [ **PAK3** ]  activated kinase PAK signaling pathway.  Ref: 12207012 J Biol Chem, 2002
- Increased spreading, Rac  [ **RAC1** ]  p21  [ **PAK3** ]  activated kinase PAK activity, and compromised cell motility in cells deficient in vasodilator stimulated phosphoprotein VASP.  Ref: 12055190 J Biol Chem, 2002
- ... p21  [ **PAK3** ]  activated kinase, an effector of Rac  [ **RAC1** ] , is required for cell motility.  Ref: 12134084 Mol Biol Cell, 2002
- Overexpression of constitutively active mutants of p21  [ **PAK3** ]  Ras, or Rac 1  [ **RAC1** ] , but not of PI 3K, was sufficient for activation of p38 mitogen activated protein kinase MAPK in cells of hemopoietic origin.  Ref: 12384416 Blood, 2002
- Evidence has been provided that direct binding of Cdc42 guanosine triphosphate and  **Rac1**  GTP to the N terminal regulatory domain of the p21  [ **PAK3** ]  activated kinase stimulates PAK activation and actin reorganization.  Ref: 12453877 Blood, 2002
- Inhibition of p21  [ **PAK3** ]  activated kinases Paks, downstream effectors of  **Rac1** , inhibited  **Rac1**  Q61L induced MT growth and retrograde flow.  Ref: 12796474 J Cell Biol, 2003
- Increased kinase activity of p21  [ **PAK3** ]  activated kinase, a specific downstream effector of activated Rac  [ **RAC1** ]  Cdc42 was also observed during the time course of infection.  Ref: 12390349 Cell Microbiol, 2002
- This study looked for the effect of 3 hydroxy 3 methylglutaryl coenzyme A HMG CoA reductase inhibitors on NADPH oxidase dependent superoxide anion production in THP 1 cells, a monocyte derived cell line, and on the translocation of p21  [ **PAK3** ]  Rac  [ **RAC1** ]  2 and p67.  Ref: 12352324 J Cardiovasc Pharmacol, 2002
- Cell attachment to the extracellular matrix induces proteasomal degradation of p21  [ **PAK3** ]  CIP1 via Cdc42  **Rac1**  signaling.  Ref: 12052868 Mol Cell Biol, 2002
- In the absence of chemoattractant, expression of constitutively active Rac  [ **RAC1** ]  stimulates accumulation at the plasma membrane of actin polymers and of GFP tagged fluorescent probes for PI, 5 P3 the PH domain of Akt and activated Rac  [ **RAC1** ]  the p21  [ **PAK3** ]  binding domain of p21  [ **PAK3** ]  activated kinase.  Ref: 12551955 J Cell Biol, 2003
- In accordance with these observations, activation of the Rac  [ **RAC1** ]  p21  [ **PAK3** ]  activated kinase PAK pathway, a crucial element in the regulation of cell spreading, is markedly enhanced in VASP cells.  Ref: 12055190 J Biol Chem, 2002
- The pathway initiated by m2 receptors involved sequential activation of Gbetagamma i3, phosphatidylinositol PI 3 kinase, Cdc42, and  **Rac1** , p21  [ **PAK3** ]  activated kinase PAK1, p38 mitogen activated protein MAP kinase, and cPLA 2, and phosphorylation of cPLA 2 at Ser 505.  Ref: 12576304 Am J Physiol Gastrointest Liver Physiol, 2003
- Here we demonstrate that plexin B1, a receptor for Semaphorin4D Sema4D, and p21  [ **PAK3** ]  activated kinase PAK can compete for the interaction with active Rac  [ **RAC1** ]  and plexin B1 can inhibit Rac  [ **RAC1** ]  induced PAK activation.  Ref: 11937491 Genes Dev, 2002
- In addition, simvastatin inhibited the translocation of p21  [ **PAK3** ]  rac  [ **RAC1** ]  2 and p67, suggesting that geranylgeranylation is required for NADPH oxidase activation.  Ref: 12352324 J Cardiovasc Pharmacol, 2002
- In brain, Shank forms a complex with beta PIX and beta PIX associated signaling molecules including p21  [ **PAK3** ]  associated kinase PAK, an effector kinase of  **Rac1**  Cdc42.  Ref: 12626503 J Biol Chem, 2003
- The p21  [ **PAK3** ]  activated kinases PAKs 1 3 are serine threonine protein kinases whose activity is stimulated by the binding of active Rac  [ **RAC1** ]  and Cdc42 GTPases.  Ref: 12676796 Annu Rev Biochem< MedlineTA>Annu Rev Biochem, 2003
- One of the effectors of  **Rac1**  and Cdc42, p21  [ **PAK3** ]  activated kinase 1, is abundant in the brain.  Ref: 12213441 Mol Cell Neurosci, 2002
- Based on upregulation of p21  [ **PAK3** ]   **rac1**  and MAPK p38alpha, activation of the MAPK pathway appears to be an early event in colonic carcinogenesis.  Ref: 12865273 Gut, 2003
- The present study aimed to elucidate the subcellular distribution of the Cdc42 and Rac  [ **RAC1** ]  activated alpha and beta isoforms of p21  [ **PAK3** ]  activated kinase in the monkey cerebral neocortex and hippocampus.  Ref: 12012157 Exp Brain Res, 2002
- RESULTS Evaluation of gene expression profiles of normal versus adenomatous colonic crypts of six different patients revealed, in general, dysregulation of up to 11% of all analysed genes total number n=588 specifically, p21  [ **PAK3** ]   **rac1**  was upregulated in four of six patients, mitogen activated protein kinase MAPK p38alpha in three of six patients, and interferon gamma receptor in three of six patients.  Ref: 12865273 Gut, 2003
- In these samples, upregulation of proliferation associated genes ras oncogene related p21  [ **PAK3** ]   **rac1**  and MAPK p38alpha as well as downregulation of apoptosis related genes FAST kinase and p53 most likely reflects specific alterations in adenomas with low grade dysplasia.  Ref: 12865273 Gut, 2003
- ATX activated Cdc42 and  **Rac1**  and increased complex formation between these small G proteins and p21  [ **PAK3** ]  activated kinase PAK.  Ref: 12482591 FEBS Lett, 2002
